# Individualized perioperative management in transoral spine surgery: a single-center cohort study evaluating surgical wound complications and wound infections

**DOI:** 10.1186/s12871-022-01673-x

**Published:** 2022-04-27

**Authors:** Vera Spatenkova, David Sila, Milada Halacova, Jan Hradil, Zdenek Krejzar, Eduard Kuriscak

**Affiliations:** 1 Neurocenter, Neurointensive Care Unit, Regional Hospital, Husova 357/10, 46063 Liberec, Czech Republic; 2 First Medical Faculty, Institute of Physiology, Charles University, Prague, Czech Republic; 3 Department of Anaesthesia and Intensive Care Medicine Third Medical Faculty, Charles University, Prague, Czech Republic; 4 Faculty of Health Studies, Technical University of Liberec, Liberec, Czech Republic; 5 Anaesthesia and Intensive Care Department, Regional Hospital Liberec, Liberec, Czech Republic; 6 Department of Clinical Pharmacology, Na Homolce Hospital, Prague, Czech Republic; 7 Neurocenter, Department of Neurosurgery, Regional Hospital Liberec, Liberec, Czech Republic; 8 Department of Neurology First Medical Faculty, Charles University, Prague, Czech Republic

**Keywords:** Surgical site infection, Transoral, Surgery, Frailty, Wound complications, Antibiotic prophylaxis, Skull base

## Abstract

**Background:**

Transoral spine surgery is specific due to both its surgical approach and the spectrum of diseases it targets. Patients with high age and elevated clinical frailty scores are often involved, and there are reports of increased risks of surgical site infection (SSI) due to extended exposures requiring maxilotomy or mandibulotomy. Our case series describes surgical wound complications under the meticulous application of individualized perioperative multimodal management.

**Methods:**

Our primary outcome was the occurrence of SSI and the secondary outcome was the occurrence of other noninfectious wound complications evaluated in 22 adult patients who consecutively underwent the transoral spine surgery from 2001 to 2018 (trauma – C2, cervical nonunion: 6 patients, 27%; tumor: 4 patients, 18%; osteomyelitis: 6 patients, 27%; other non-traumatic cases: 6 patients, 27%). Structuralized data comprising parameters related to nosocomial infections after spine surgery were continuously processed and put into specialized database of preventive multimodal nosocomial infection control protocol that was used as a main source of analyzed parameters. The mean age of studied cohort was 54.9 $\pm$ 15.5 years, with 68% males, mean body mass index (BMI) 24.9 $\pm$ 5.22, and the mean clinical frailty score was 2.59 $\pm$ 1.07. There were 7 patients (32%) who only had the transoral approach and 15 patients (68%) having this approach followed by additional posterior approach. We observed SSI from all wound complications for up to one year after surgery.

**Results:**

There were 4 (18%) superficial wound complications from transoral approach, but none of them were infected. We had 2 patients (13%) with deep wound infections after subsequent posterior approach, but only one (4.5%) was classified as SSI.

**Conclusions:**

We describe the wound complications and the incidence of SSI in a series of 22 patients after the transoral surgery. Considering the average values of the clinical frailty score reaching 2.59, American Society of Anesthesiologists score of 2.73, and the BMI of 26.87, the transoral spine surgery did not seem to be a considerable risk for SSI in the analyzed cohort, provided preventive perioperative multimodal management is properly individualized and followed.

## Introduction

Transoral (TO) spine surgery is specific due to both its surgical approach and the spectrum of diseases it targets [1, 2]. Patients with high age and elevated clinical frailty scores are often involved and there are reports of increased risk of surgical site infection (SSI) mainly due to perioperative considerations, technical aspects pertaining the TO approach, prolonged surgery and more complex techniques involving maxilotomy or mandibulotomy [3]. The perioperative stress is often increased by a necessity for an additional surgery—a stabilization via posterior approach. The preventive perioperative multimodal management has an important role in the reduction of adverse events of SSI. A careful and individualized perioperative strategy is necessary and represents an indicator of operation care quality.

The transoral approach was introduced by Kanavel [4] in 1917 as a novel approach to reach the upper cervical vertebrae (C1-C2). Surgical site infections represent an unavoidable risk in any surgery, but in the TO spine surgery, the risk of SSI is increased due to the food intake during the postoperative period and the possibility of perioperative wound contamination. The preventive multimodal wound control protocol relies on a hygienic regime in the operative and postoperative periods and assumes a correct antibiotic prophylaxis [5, 6]. An inappropriate (often excessive) use of antibiotics worsens the epidemiological status and leads to the rise of multidrug resistant bacteria.

In this study we focused on the description of:

1. the important aspects and parameters of individualized perioperative management (duration of mechanical ventilation, use of endotracheal and tracheostomy tubes, nasogastric tubes, arterial and venous catheters, type of nutrition and drug prophylaxis)
2. the risks of the TO approach for odontoid osteomyelitis compared to non-infectious pathologies (including the SSI risks for subsequent posterior approach)
3. the recurrent osteomyelitis after surgery
4. the influence of the extent of TO surgery on the SSI
5. the time interval between the TO surgery and the additional surgery via posterior approach.

The aim of this case series study is the characterization of surgical wound complications. We focused on the primary outcome which was the evaluation of the incidence of SSI after the transoral surgery that adheres meticulously to a modern perioperative preventive multimodal wound control protocol individualized according to the patient status. The secondary outcome we were interested in was the occurrence of noninfectious surgical wound complications (dehiscence, hematoma, secretion, liquorrhea, metalwork prominence, etc.).

## Materials and methods

### Data source and data collection

The prospective database of preventive multimodal nosocomial infection control protocol of the Neurointensive Care Unit, belonging to the Regional Hospital Neurocenter—one of the country’s spinal surgery centers—is maintained since 2001. It contains prospective data related to all parameters collected with respect to monitored nosocomial infections in our NICU, and other parameters related to patients’ health status. This study analyses a series of 22 patients who underwent the TO surgery consecutively from 2001 to 2018, with no patients excluded from this study. Our TO surgery patients have been recruited from all over the republic. We classified SSI according to the location of infection into superficial (involving skin and subcutaneous tissue), deep (deep soft tissue as fascial and muscle layers), and organ/space (part of the body deeper than the fascial/muscle layers that is opened or manipulated during the operative procedure). The patient status was classified mainly by intensive and emergency medicine scores and indexes, namely, the American Society of Anesthesiologists (ASA) score, the Acute Physiology and Chronic Health Evaluation (APACHE) score, the Therapeutic Intervention Scoring System (TISS), and by the three types of patient frailty scoring systems (Frailty index, Frailty index-11 and Clinical frailty score) related to postoperative morbidity and mortality.

The demographic data, history of diseases and spine diagnosis are listed in Table 1.

**Table 1:** Demographic and clinical data of the patients

**Parameter (*N* = 22)**	**Unit**	%	Minimum	Maximum	Mean	Standard deviation	Median
Age	year		23	77	54.9	15.5	60
Female	pts	31% (7)					
Male	pts	68% (15)					
Weight	kg		41	105	72.0	17.1	73.0
Body Mass Index			23.8	29.6	26.8	4.4	26,4
Spine diagnoses							
Trauma	pts	27% (6)					
Non-union of the dens axis fracture	pts	5					
Tumor	pts	18% (4)					
Chondroma	pts	2					
Metastasis	pts	1					
Other non-traumatic	pts	27% (6)					
Rheumatoid arthritis	pts	2					
Osteomyelitis	pts	27% (6)					
Localization							
C 1–2	pts	68% (15)					
C 2	pts	27% (6)					
C 2–4	pts	4% (1)					
Coronary artery disease	pts	4% (1)					
Arterial hypertension	pts	54% (12)					
Bronchial asthma	pts	9% (2)					
COPD	pts	4% (1)					
Chronic renal failure	pts	9% (2)					
Hepatopathy	pts	13% (3)					
Gastroduodenal ulcer	pts	9% (2)					
Diabetes Mellitus	pts	13% (3)					
Smoking	pts	18% (4)					
Ethylism	pts	13% (3)					
Frailty index			0	5	1.27	1.35	1
Frailty index 11			0	0.45	0.11	0.12	0.09
Clinical frailty score			1	5	2.59	1.07	3
Stay in the ICU			0	35	8.41	7.74	6.50
Total our hospital stay			1	73	23.45	16.99	18.50

*N* Number, *C* Cervical, *COPD* Chronic Obstructive Pulmonary Disease, *ICU* Intensive Care Unit

The type of operation approach, duration of surgery, instrumented fixation, blood loss, transfusions, drainage, ASA score, are seen in Table 2. Postoperative neurocritical care procedures and related details (mechanical ventilation, endotracheal tube, tracheostomy tube, arterial, central and urine catheters, gastrointestinal tube, body temperature, use of corticoids, ulcer prophylaxis, nutrition), associated health complications, TISS and APACHE II scores, are all described in Table 3. Particular numbers of patients and durations in days listed in Table 3 show our individualized approach regarding the postoperative care, demonstrating our decisions respecting individual conditions of our patients before the surgery, the type of surgery, and their conditions after the surgery. We individually considered these conditions and made decisions regarding:1) the use of mechanical ventilation using endotracheal tube or tracheostomy tube, 2) urine catheters, 3) peroral vs. enteral (nasogastric tube) vs. parenteral nutrition (further parameters are listed in Table 3 and also in Table 4 which describes the use of antibiotics). Laboratory data (preoperative and postoperative blood and biochemical parameters like hemoglobin, hematocrit, leukocytes, glycemia, albumin, proteins, lactate, C-reactive protein) are in Table 5.

**Table 2:** Characteristics of the operations

**Parameter**	**Unit**	**N**	**%**	Minimum	Maximum	Mean	Standard deviation	Median
ASA score				1	4	2.73	0.69	3
Day of hospitalization	day	22		1	21	6.86	5.75	4.5
Time of operation	minutes			25	1320	339	288	325
Biopsy before	pts		13% (3)					
Transoral operation	pts	22	31% (7)					
Transoral and posterior	pts		68% (15)					
Consecutive	pts		14					
Biopsy only	pts		0% (0)					
Odontoid resection	pts	22	72% (16)					
Extensive approach	pts		27% (6)					
Posterior approach	pts							
Graft only	pts		6% (1)					
C1-C2 Magerl, Gallie	pts	15	26% (4)					
C1-C2 Harms	pts		20% (3)					
Occipito-cervial fusion	pts		46% (7)					
Graft								
Without	pts		13% (2)					
Bone substitute	pts	15	6% (1)					
Autologous free	pts		33% (5)					
Autologous fix	pts		46% (7)					
Drainage Posterior	pts		100% (15)					
Suction drainage	pts		14					
	number	15		1	2	1.50	0.50	1.50
	day			2	3	2.14	0.35	2.00
Gravity drainage	pts		2					
Blood loss	ml	22		50	4000	1305	1333	800
Transfusions	pts		31% (7)					

*N* Number, *ASA* American Society of Anesthesiologists

**Table 3:** Characteristics of the postoperative period

**Parameter (*N* = 22)**	**Unit**	%	Minimum	Maximum	Mean	Standard deviation	Median
Admission TISS			52	58	55.10	1.57	55
Admission APACHE II			2	15	8.90	3.69	9.50
Mechanical ventilation	pts	45% (10)					
Time	day		1	17	3.90	4.78	1.50
Endotracheal tube	pts	13% (3)					
Time	day		1	1			
Tracheostomy tube	pts	54% (12)					
Time	day		2	85	13.58	22.58	4.00
Artery catheter	pts	59% (13)					
Time	day		1	12	4.50	2.87	4.50
Radialis	pts	12					
Central venous catheter	pts	31% (7)					
Time	day		2	19	9.17	5.27	7.50
Subclavia	pts	5					
Femoralis	pts	2					
Urine catheter	pts	90% (20)					
	day		1	33	8.05	7.98	4.00
Nasogastric tube	pts	81% (17)					
	day		1	15	5.06	3.24	5.00
Body temperature (max)			33	39.5	37.3	2.05	38.00
Complications							
Delirium	pts	9% (2)					
Respiratory	pts	9% (2)					
Hemodynamics	pts	40% (9)					
Acute kidney injury	pts	18% (4)					
Dysphagia	pts	27% (6)					
Enteral nutrition	pts	68% (15)					
	day		2	11	4.46	2.44	4.00
Parenteral nutrition	pts	22% (5)					
	day		1	28	9.40	9.77	7.00
Insulin	pts	40% (9)					
Ulcer prophylaxis	pts	72% (16)					
Corticoids	pts	68% (15)					
Dexamethasone	pts	3					
Methylprednisolone	pts	6					
Hydrocortisone	pts	5					

Parameters in the table represent numbers of patients or days that demonstrate either the size of patient subgroups or the duration of complications in days, as well as our decisions respecting the individual approach to our patients during the perioperative period

*N* Number, *TISS* Therapeutic Intervention Scoring System, *APACHE* Acute Physiology and Chronic Health Evaluation

**Table 4:** Antibiotic prophylaxis in the non-infection transoral surgery

**Parameter (*N* = 16)**	**Unit**	%
Antibiotic prophylaxis	pts	72% (16)
Operation doses	pts	25% (4)
Day 1	pts	6% (1)
Day 2	pts	6% (1)
Day more than 2	pts	62% (10)
Antibiotic 1	pts	37% (6)
Antibiotic 2	pts	56% (9)
Antibiotic 3	pts	6% (1)
Cefazolin	pts	75% (12)
Amoxicillin clavulanate	pts	56% (9)
Clindamycin	pts	6% (1)

*N* Number

**Table 5:** Laboratory examination

Parameter	Unit	Reference range	Before operation mean $\pm$ SD	After operation mean $\pm$ SD	
Haemoglobin	g/l	135–175	129.45 $\pm$ 18.35	93.05 $\pm$ 22.09	The lowest value
Hematocrit		0.4–0.5	0.38 $\pm$ 0.06	0.28 $\pm$ 0.07	The lowest value
Leukocytes	10^9^/l	4–10	7.37 $\pm$ 2.84	17.31 $\pm$ 4.79	The highest value
Albumin	g/l	35–52	34.54 $\pm$ 5.47	26.42 $\pm$ 7.19	The lowest value
Protein	g/l	66–87	70.50 $\pm$ 4.70	43.91 $\pm$ 26.00	The lowest value
C-reactive protein	mg/l	0–5	11.54 $\pm$ 14.55	94.44 $\pm$ 75.20	The highest value
Glycemia	mmol/l	4.1–5.6	6.04 $\pm$ 2.02	10.6 $\pm$ 3.44	The highest value
Lactate	mmol/l	0.36–0.75		2.76 $\pm$ 1.51	On admission ICU
				3.10 $\pm$ 2.03	The highest value

*SD* Standard Deviation, *ICU* Intensive Care Unit

In the study we also included parameters related to an overall health status of the patient represented by the Frailty Index [7], Frailty Index 11 [8] and Clinical Frailty score [7] as seen in Table 1.

### Study design

The approval to process the data from our preventive multimodal nosocomial infection control protocol database was issued by the Hospital Ethics Committee (ref. number of approval EK27). All participants gave written informed consent prior to all measurements and agreed with publication. All methods were performed in accordance with the relevant guidelines and regulations.

As mentioned, the TO surgery cohort consisted of a series of 22 patients, with the mandibular or maxillary split indicated in 6 (27%) patients. In the subgroup of 15 probands, the TO approach was supplemented by a subsequent dorsal fixation operation – the posterior surgery route.

Patients were divided into two subgroups according to the extent of transoral resection:

1. subgroup of simple transoral odontoid resection and
2. subgroup of more extensive approach with the split of either maxilla or mandibula.

The data concerning the posterior approach (concerning 15 patients) fell into 4 subgroups:

1. dorsal interlaminar grafting only
2. transarticular C2-C1 Magerl fixation supplemented by interlaminar Gallie fusion
3. C1-C2 Harms fixation and fusion
4. occipito-cervial fusion

Every preventive multimodal wound control protocol comprises of a correct antibiotic prophylaxis with an emphasis on dosage and timing of administration before and during the operation. Cefazolin was our first choice of antibiotic, with Clindamycin administered in case of allergy to beta-lactam antibiotics. Cefazolin was administered 30–60 min before surgery (namely before the incision, 2 g if the body mass was less than 100 kg, otherwise 3 g), and re-administered, if the surgery lasted longer than 4 h, or if the blood loss was bigger than 1.5 L. The dose of Clindamycin was 600 mg, administered 60 min before surgery, and repeated if it was longer than 6 h (for body weight above 100 kg the dose was 900 mg). The prolonged use of antibiotics after the operation was reduced as much as possible. We adhered the antiseptic regime of surgery approach, wound care, single-use products, closed systems, the minimum duration of surgery, minimal and only necessary disconnection of used port systems.

Surgical site infections were defined according to 1) clinical symptoms, 2) bacterial pathogens, 3) imaging methods, 4) biochemical and hematological laboratory tests. SSI were followed and evaluated up to one year after the surgery.

### Statistical analysis

The statistical analysis was done in Microsoft Excel. We evaluated parameters of descriptive statistics. We calculated minima, maxima, medians, means, standard deviations (SD), frequencies and percentages of evaluated variables.

## Results

Regarding the presented cohort of patients undergoing the TO surgery, there were 4 (18%) patients with transoral superficial wound complications (2 patients with tumor, one with traumatic etiology, one with non-traumatic etiology). However, none of those patients had infection of the wound. The noninfectious complications from the TO approach were the dehiscences of the pharyngeal mucosa that were solved with a conservative treatment and concerned 3 nonsmoker patients with BMI 21.8, 24.2, and 26.2. They had the nasogastric tube for 5, 8, 14 days, and started to eat orally once the dehiscence was cured. Noninfectious wound complication of the fourth patient was the metalwork prominence which caused serious dysphagia and was solved surgically by reoperation on 27^th^ day (that patient was cachectic, ethylic and smoker, with BMI 13.9). None of those four patients had pathological cultivation, all had tracheostomia, all received Amoxicilin clavulanat as profylaxis, and three of them were on mechanical ventilation.

Two deep wound infections were registered after the posterior approach surgery. The first wound infection developed in the 69-year-old lady with the frailty index score 3, after the TO and subsequent posterior spine surgery lasting 440 min altogether, with a blood loss of 1000 ml (it was performed due to nonunion of the fracture of axis vertebra). This infection occurred 34 days after the surgery, so it was classified as SSI according to the SSI guidelines [9]. It was caused by Bacteroides species and Peptostreptococcus species according to microbiological testing. This deep infection was found in a surgical wound with healed skin suture underneath which a subcutaneous palpable pus collection (containing approx. 30 ml of thick smelling pus) was found and punctured. That patient had a higher risk of SSI because she had tonsillitis with fevers reaching 39 °C, which was treated with Amoxicilin clavulanat antibiotics before this SSI was found.

The second deep wound infection was observed in a 63-year-old man with the frailty index score 3, after the TO and subsequent posterior spine surgery lasting 1320 min both, with a blood loss reaching 4000 ml. This operation was performed due to a C2 chordoma extending into surrounding tissues, and the infection appeared 6 months after the surgery as a purulent fistula and recurrence of a chondroma. Pathogens were identified as Staphylococcus aureus and Escherichia coli. However, that infection was not classified as SSI according to the CDC guidelines [9] since it had not appeared within a 90-day period after surgery. Thus, the overall incidence of SSI after the TO approach reached 4.5% (in one of 22 patients) in our cohort.

Table 1 shows the spectrum of spine diagnoses involved in this study, frailty scores and indexes, patient comorbidities and demographics. In Table 2 are seen details about performed surgery, blood loss and transfusions. The postoperative care details that are reflecting our individualized postoperative approach that is taking into account preoperative as well as postoperative conditions and complications influencing our decision regarding the appropriate approach for each patient (duration of mechanical ventilation, use of the endotracheal tube, tracheostomy tube, nasogastric tube, type of nutrition and pharmacotherapy, etc.), are listed in Table 3. Detailed information on antibiotic prophylaxis are seen in Table 4. Table 5 contains information on blood examinations before and after surgery.

## Discussion

Surgery via transoral approach is considered to have an inherently higher risk of SSI and a higher number of complications during the postoperative period. This is mainly attributed to a specific surgical route through the oral cavity and less familiar anatomy associated with it [10, 11]. These appear to be factors limiting a wider adoption of this technique, however, regarding the incidence of SSI, there is some data indicating that the TO approach does not necessarily increase it when compared to the posterior approach, at least concerning the 30-day follow-up period after surgery [12].

The gradual renaissance of the TO approach is linked not only with newer operative technologies (e.g., anterior fixation via the TO approach [13] or robot-assisted TO [14]), but also with a deeper understanding of preventive and therapeutical measures. These include a standardized preventive multimodal wound control protocol that is concentrating on the preoperative, perioperative and postoperative phases, being properly individualized to respect the health status of the patient, an approach that is playing a pivotal role in the success of the treatment. The incidence of SSI is thus considered a significant quality indicator of surgery and individualized perioperative care.

In our prospective database of preventive multimodal nosocomial infection control protocol, we analyzed 22 patients who underwent the TO surgery during the reference period from 2001 to 2018. Both infectious and non-infectious pathologies were included in the study. Our results show one SSI developed after the TO surgery. There were 4 superficial wound complications (3 mechanical dehiscences of the pharyngeal mucosa and 1 metalwork prominence) after the TO approach, however, with no pathological cultivation, and this complication was not detected in patients with osteomyelitis surprisingly.

Our data shows 4.5% incidence of SSI after the TO surgery, which means that the SSI developed in just one patient whose spine was fixed anyway with the consecutive posterior surgery—a value comparable with other monocentric studies focusing on the SSI incidence after TO surgery. In a study by Yin et al. [3], the incidence reached 3.5% (172 patients), and in [12] it was 1.79% (56 patients).

TO surgery often requires subsequent posterior spine stabilization. This can be done in a single session (in case there is a significant loss of spinal stability after TO surgery), or it can be postponed and performed separately under more favorable conditions. The combination of TO surgery and consecutive fixation with posterior approach was performed in 15 patients, with 14 patients undergoing single-stage surgery, leading to longer total operation times (mean 339 min). Although the duration of surgery is known to be a significant factor increasing the incidence of SSI [15, 16], it is very likely that such surgery durations did not increase the incidence of SSI in our patients.

Having just one SSI in our cohort of 22 patients also reflects the indication criteria (see Table 1), and a deliberate selection of patients elected for TO surgery in our hospital. A proper selection of patients, based on the complex conciliar discussion that is considering possible prognosis and outcomes, and the meticulous preoperative preparation, are of the utmost importance for a long-term success of TO surgery. Taking into account the extent and the duration of this type of surgery, only patients with responsibly chosen TO indication and in good health conditions will have acceptable postoperative complications and low mortality rate. In the reported cohort, there were no highly polymorbid patients: coronary artery disease—1 patient, COPD—1 patient, chronic renal failure—2 patients, diabetes mellitus—3 patients. 12 patients had arterial hypertension and there was no substantial obesity (mean BMI 26.87). The ASA score (mean 2.73) and the clinical frailty score (mean 2.59) reflect our deliberate selection of patients undergoing the extensive TO surgery. We included frailty scores (see Table 1) because many clinicians think this scale has a good predicting value and commonly outperforms other measures of comorbidity and risk of death—or outcomes of serious health conditions, including complications from surgery and corresponding postoperative mortality and morbidity [7, 8].

Standardized wound care is an irreplaceable part of the multimodal wound control protocol. This includes the individualized preoperative assessment of risks and the intended extent of surgery. To the most important steps belong the individualized decisions about transoral/transnasal intubation versus tracheostomy (12 patients, 55%), which is influencing the incidence of SSI, favoring the latter as more advantageous in this regard. Also, the form of nutrition after the TO surgery must be responsibly assessed since the nasogastric line is placed near surgery wounds and could increase the incidence of SSI during the postoperative period. We used enteral nutrition in 15 patients (68%), and only 5 patients required parenteral nutrition (5 patients, 23% patients).

The wound care is rather atypical in patients undergoing the TO surgery and consists of proper preoperative preparation, meticulous technique of closure and proper postoperative regime. Any contact between the wound and the tubes (endotracheal, nasogastric, etc.) should be avoided. If this is not feasible, changes must be made in the position of the tubes to prevent decubitus. We observed 3 cases of superficial pharyngeal dehiscence with negative bacteriological cultivations. These defects often involve only part of the suture and tend to heal quickly by epithelization in the absence of bacterial superinfection. There was only one SSI despite the 6 cases of osteomyelitis and the use of corticoids in 15 patients (68%).

The most common postoperative complication seen was a hemodynamic instability requiring vasopressors (9 patients, 41%). The mean lactate level after surgery was 2.76 mmol/L, with a maximum value reaching 3.1 mmol/L.

One of the most important parts of the protocol is a proper antibiotic policy. The unique nature of TO surgery prevented us from adhering to a protocol used in general spine surgery (one dose preoperatively and intraoperative administration only – that was applied only in 4 cases). There were 2 patients receiving antibiotics for up to 48 h postoperatively and 10 patients (63%) who received another dose of antibiotics. Prolonged prophylaxis was required in 3 cases of superficial dehiscence, but some of reported patients (namely those with osteomyelitis) required prolonged antibiotic therapy. The cefazolin was used as a first-choice prophylactic antibiotic (12 patients, 75%), followed by Amoxicillin clavulanate (9 patients, 56%).

Our study has several limitations. Despite a 17-year span, we only had 22 patients undergoing the TO surgery in our neurocenter. Moreover, our population sample had a low number of polymorbid and obese patients, low frailty score and low number of smokers.

However, the study is monocentric, analyzing our own cases of relatively rare TO surgery, known to be much less frequent compared to other types of spine surgery.

## Conclusions

We analyzed wound complications and the incidence of SSI in a series of 22 patients undergoing the transoral surgery. We report the ASA, APACHE and TISS scores as well as the three frailty measures correlating with the patient health status. Having only one SSI, and taking into account evaluated patient score levels, we would like to conclude that the transoral spine surgery did not seem to be a considerable risk for SSI in our cohort, assuming an individualized perioperative multimodal preventive management and adequate indication criteria for TO surgery are met.

## Data Availability

The datasets generated and/or analysed during the current study are not publicly available due due to the anonymity of the participants but are available from the corresponding author on reasonable request.

## Abbreviations

APACHE: Acute Physiology and Chronic Health Evaluation
ASA: American Society of Anesthesiologists
C: Cervical
COPD: Chronic Obstructive Pulmonary Disease
ICU: Intensive Care Unit
N: Number
NICU: Neuro-Intensive Care Unit
SSI: Surgical Site Infection
SD: Standard Deviation
TISS: Therapeutic Intervention Scoring System
TO: Trans-Oral
